# Metacognition, Desire Thinking, and Problematic Social Media Use: Investigating Effects of Active and Passive Engagement in Facebook and Instagram Users

**DOI:** 10.1111/sjop.13119

**Published:** 2025-04-30

**Authors:** Andrew Allen, Geraldine Sanders‐Westerhof, Azin Khodadadi, Lee Kannis‐Dymand

**Affiliations:** ^1^ University of the Sunshine Coast School of Health Sippy Downs Queensland Australia

**Keywords:** active social media use, desire thinking, metacognition, passive social media use, problematic social media use

## Abstract

The current study aimed to improve the understanding of cognitive and metacognitive processes underlying problematic social media use (PSMU) by comparing active and passive user engagement and contrasting Facebook and Instagram users. The metacognitive model of desire thinking (MCMDT) was utilized to explore these distinctions within a community sample of 337 participants (64% female, *M*_*age*_ = 36.10). Participants completed self‐report measures to assess their metacognitive processes, desire thinking patterns, and social media engagement. The MCMDT demonstrated robust fit across all scenarios, with the most optimal fit for Instagram (*χ* ^2^(3) = 0.850, *p* = 0.838, CFI = 1.000, RMSEA = 0.000, SRMR = 0.0062, variance explained 59.4%), and the least optimal fit for passive users (*χ* ^2^(3) = 1.151, *p* = 0.765, CFI = 1.000, RMSEA = 0.000, SRMR = 0.0206, variance explained 45.9%). The initiation and elaboration stages of the MCMDT were supported, but the pathological amplification stage was not. As expected, active users showed better model fit than passive users, and Facebook users had more pronounced verbal perseveration (VP) activation during the elaboration stage. Contrary to expectations, Instagram did not display more pronounced imaginal prefiguration (IP) activation than Facebook. These results enhance understanding of the cognitive and metacognitive processes driving PSMU, which may contribute to developing targeted interventions. Future research should investigate how VP and IP interact with and influence the later stages of the MCMDT of desire thinking across various populations and contexts.

**Summary**

- This study explored how metacognition—the way people think about their thoughts—might explain why some people struggle to control their use of Facebook and Instagram.
- Findings suggest that Facebook users were more likely to get caught in repetitive thinking patterns, while Instagram users demonstrated less problematic mental imagery than expected.
- Overall, how people reflect on and manage their thoughts about social media influences problematic social media use, but patterns differ depending on the platform and style of use.

Social media is an indispensable component of contemporary living, transforming engagement in various activities, such as social interactions, networking, romantic connections, and business pursuits across multiple digital platforms (Aichner et al. 2021). The adoption of social media continues to expand, with followers and engagement duration steadily increasing. In January 2025, 63.9% of the global population used social media (5.24 billion), with the average user engaging in 6.8 different social media platforms each month (DataReportal 2025). Major platforms like Facebook, YouTube, WhatsApp, Instagram, WeChat, and TikTok dominate the market, with Facebook claiming 2.96 billion users and Instagram hosting 2 billion users monthly (Dreamgrow 2025). While social media typically serves as an enjoyable pastime, it can also impact mental health and well‐being.

Since the emergence of leading social media platforms over the past two decades, there has been a significant surge in research exploring the potential mental health consequences of social media usage. Initial investigations aimed to identify aspects of mental health, such as well‐being, anxiety, and depression, that were affected. Despite the efforts of these early, primarily cross‐sectional studies, the results were inconclusive (for reviews, see Andreassen 2015; Pantic 2014). As research methodologies advanced, experimental studies began to establish causal links, revealing minor yet notable decreases in well‐being (e.g., Vanman et al. 2018). Similarly, longitudinal research discovered persistent negative correlations with well‐being (e.g., Shakya and Christakis 2017). Concurrently, meta‐analyses confirmed this modest yet significant negative association between problematic use and well‐being, depression, anxiety, and stress (e.g., Duradoni et al. 2020). Kross et al. (2021) offer a comprehensive overview of social media use and well‐being, including distinctions between active and passive engagement, and a shift towards investigating moderating factors and underlying psychological mechanisms.

## 1 Factors Influencing Social Media's Impact on Mental Health

Investigating the impact of social media on mental health necessitates a nuanced approach with three factors being plausibly relevant due to their influence on mental health: (1) the nature of user engagement (either active or passive), (2) the unique characteristics of different social media platforms, and (3) the lack of a standardized definition for problematic social media use (PSMU). Patterns of social media use, particularly active and passive engagement, may influence cognitive and metacognitive processes differently (Akbari et al. 2024). Active engagement, involving intentional interactions such as liking, sharing, commenting, or creating content (Verduyn et al. 2015), is generally associated with positive mental health outcomes (Caselli and Spada 2010; Verduyn et al. 2015). Active use that encourages the formation, evaluation, and revision of information may rely on skills such as reasoning, self‐monitoring, and strategic thinking (Nam and Hwang 2021; Wu et al. 2014). Conversely, passive social media use, characterized by less direct interactions like viewing pictures, videos, or reading discussions (Verduyn et al. 2015), has been associated with negative mental health outcomes such as depression and anxiety (Frison and Eggermont 2020; Verduyn et al. 2015, 2022; Vogel et al. 2014). This mode of engagement may imply lower cognitive involvement, correlating with less intentional cognitive engagement while using social media.

Platforms like Facebook and Instagram, through their unique attributes, may influence engagement toward distinct cognitive processes (Facebook, n.d.). For instance, the visually driven nature of Instagram may favor the engagement of cognitive processes related to mental imagery. In contrast, Facebook's narrative‐rich environment could foster more language‐based cognitive processes, leading to an ongoing mental dialogue. Such differential engagement could elucidate how various platforms distinctly influence mental health. Indeed, both Instagram and Facebook use have been associated with negative psychological outcomes, with mixed findings. Instagram use has been associated with negative body image and low self‐esteem (Brown and Tiggemann 2016), while Facebook use is more commonly associated with negative social comparison and low mood (Appel et al. 2016). However, other research suggests that Facebook use is also associated with low self‐esteem (Błachnio et al. 2016), and comparative studies indicate no significant differences in depressive symptoms between Facebook and Instagram users (Błachnio et al. 2016; Limniou et al. 2022).

Finally, the field of study faces a significant challenge without a unified definition for PSMU. A growing body of research positions PSMU within the context of behavioral addiction, given its shared attributes with substance addiction and gambling disorder (Bányai et al. 2017; Escobar‐Viera et al. 2018; Griffiths 2005; Hawi and Samaha 2017; Hou et al. 2019; Shensa et al. 2018). PSMU, as a behavioral addiction, is typified by a loss of control, salience, mood modification, tolerance, withdrawal symptoms, conflict, and relapse (Griffiths 2005). Conceptualizing PSMU as a behavioral addiction allows for the application of established models to investigate cognitive and metacognitive processes. Like other behavioral addictions, PSMU involves perseverative thought patterns, craving, and maladaptive metacognitive beliefs, making these processes central to its development and maintenance (Benucci et al. 2024; Casale et al. 2018; Fioravanti et al. 2024).

## 2 The Role of Desire Thinking in Addictive Behaviors

‘Desire thinking’ is a cognitive mechanism intrinsic to addictive behaviors that has increasingly drawn research attention (Allen et al. 2017, 2021; Bonner et al. 2022; Brandtner and Brand 2021; Byrne et al. 2022; Mansueto et al. 2019; Solem et al. 2021; Thomas et al. 2020). Defined as a voluntary, conscious, perseverative, and target‐focused cognitive process, desire thinking comprises two components: imaginal prefiguration (IP; i.e., the generation of mental imagery or visualization of prospective scenarios) and verbal perseveration (VP; i.e., the propensity to reiterate words, phrases, or thoughts; Caselli and Spada 2010, 2011). When desire thinking becomes persistent and dysregulated, it may intensify intrusive thoughts and emotions, ultimately contributing to the development and maintenance of addictive behaviors (Caselli and Spada 2015).

Research has shown that desire thinking is a predictive factor for both substance addictions like nicotine and alcohol dependence (Mansueto et al. 2019; Solem et al. 2021) and behavioral addictions, encompassing compulsive sexual behavior, compulsive shopping, gambling, and online gaming alongside problematic Facebook use, internet use, pornography use, smartphone use, and PSMU (Akbari et al. 2023; Allen et al. 2017; Awad et al. 2022; Brandtner and Brand 2021; Mansueto et al. 2019; Marino et al. 2023; Sharifi Bastan et al. 2022; Solem et al. 2021). Moreover, recent research has highlighted the distinct roles of IP and VP. While the strength of the association between IP and addictive behaviors does not appear to be moderated by the subtype of addiction, VP seems to be more strongly related to substance addiction than non‐substance addiction (Mansueto et al. 2019). In addition, some studies have found that other factors (e.g., addictive behavior history, cognitive processes) may moderate the effects of IP and VP. For example, Albery and Spada (2021) found that IP directly affects drinking behaviors, whereas past behaviors significantly moderate the effect of VP on drinking behavior. Similarly, Awad et al. (2022) found that VP and IP were mediated by different factors in their association with social media use disorder. The development and maintenance of dysregulated desire thinking may be further influenced by metacognitive processes (Caselli and Spada 2011, 2013; Spada et al. 2013).

## 3 The Metacognitive Model of Desire Thinking

The metacognitive model of desire thinking (MCMDT), developed by Caselli et al. (Caselli and Spada 2011, 2013, 2015; Spada et al. 2013), advances the foundational work of Spada et al. (2008) by offering a nuanced conceptualization of how desire thinking can become dysregulated and persistent. Indeed, perseverative thinking that sustains problematic behavior reflects Cognitive Attentional Syndrome (Spada et al. 2013; Wells, 2009). However, the model delineates that the influence of metacognitive processes on problematic use is not inevitable but contingent on their management and distinctive characteristics.

The model has two primary phases. The initiation stage starts with internal or external cues like boredom or social media notifications. These cues can generate thoughts about the desired target, potentially activating positive metacognitions about desire thinking (PMDT) linked to social media use. Such positive metacognitions might include beliefs that engaging in desire thinking, such as fantasizing about potential social interactions on these platforms, aids in diverting attention away from negative emotions. For example, “using Facebook will improve my mood by making me feel more connected to my friends” (Caselli and Spada 2015).

**FIGURE 1:**
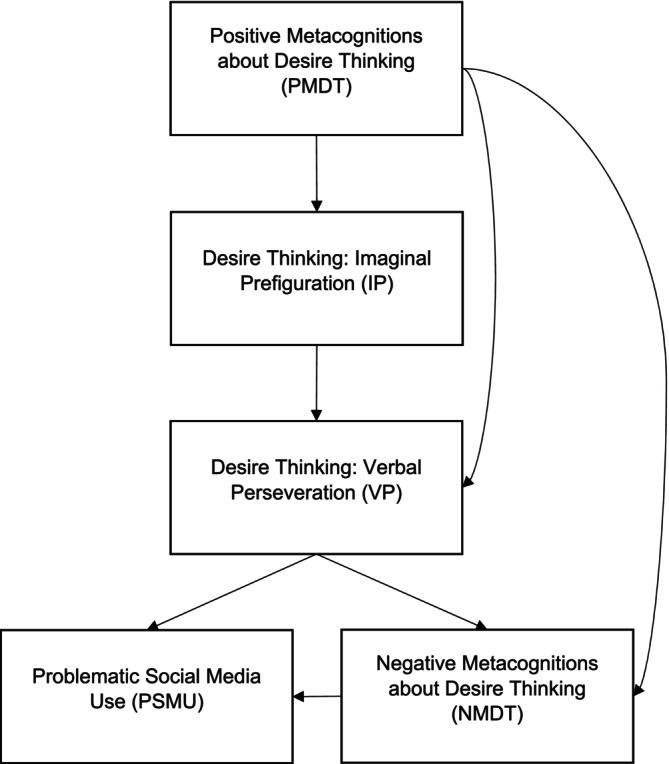
Hypothesized metacognitive model of desire thinking and PSMU.

After the initiation stage, the elaboration stage follows, marking the onset of desire thinking. This phase engages the cognitive processes of IP and VP, which aid in sustaining and intensifying the desire. Consequently, an increasingly detailed mental image or narrative about the desired activity may evolve. Overindulgence in the unregulated elaboration of desire can initiate negative metacognitions, including beliefs such as “I can't stop thinking about what I might be missing out on social media.” The development of these negative metacognitions serves as a warning sign of a pathological amplification of desire thinking (Caselli and Spada 2015; Spada et al. 2013). For a visual representation of the model, refer to Figure 1.

Increasingly, research has demonstrated empirical support for the MCMDT in both clinical and subclinical groups. Foundational studies by (Caselli and Spada et al. 2013, 2015) demonstrated a good model fit across the clinical populations of alcohol use, gambling, problematic internet use, and smoking. Subsequent studies have validated the model with problematic pornography use (Allen et al. 2017, 2021), problematic gaming (Bonner et al. 2022; Byrne et al. 2022), and binge eating (Spada et al. 2015). Each study utilized the fundamental MCMDT, with minor adjustments, including the inclusion of negative affect (Allen et al. 2017, 2021), motives (Bonner et al. 2022), the Proteus Effect (Byrne et al. 2022), and psychopathology symptoms like anger and anxiety (Allen et al. 2021; Bonner et al. 2022). These adjustments highlight the applicability of the MCMDT across various behavioral addictions, including those related to social media use.

## 4 The Present Study

Utilizing the MCMDT, this study explores cognitive and metacognitive processes among social media users, focusing on engagement type (active versus passive) and platform‐specific variations (Facebook and Instagram). Active engagement might stimulate stronger cognitive and metacognitive processes, thereby strengthening the direct and indirect pathways of the MCMDT. Additionally, the narrative‐intensive environment of Facebook could provide cues that enhance VP, whereas Instagram's emphasis on visual content might stimulate IP. By improving understanding of the underlying cognitive and metacognitive processes, this study ultimately seeks to contribute to the development of interventions to reduce problematic use.

## 5 Hypotheses

The present study examines the following hypotheses:

Hypothesis 1 *The MCMDT will show an adequate statistical fit for a community sample of social media users*.

Hypothesis 2a *The MCMDT will demonstrate an adequate statistical fit for both active and passive social media users*.

Hypothesis 2b *The direct and indirect pathways in the MCMDT are predicted to exhibit greater strength and significance among active users*.

Hypothesis 3a *The MCMDT will demonstrate an adequate statistical fit for both Facebook and Instagram users*.

Hypothesis 3b *Facebook users are anticipated to demonstrate more pronounced VP activation, while Instagram users are expected to display enhanced IP activation*.

## 6 Methods

### 6.1 Participants

Individuals aged 18 years or older were eligible to participate in the study with no exclusion criteria other than a proficient understanding of English and access to a computer. The final sample consisted of 337 participants (64% female) aged 18 to 74 (*M* = 36.10, SD = 11.18). Facebook was the preferred social media platform for 131 participants, while Instagram was the preferred platform for 133 participants.

### 6.2 Measures

Participants completed an online questionnaire with 51 self‐report items. Nine questions developed for this study covered demographics, active social media accounts, and usage. Participants also reported their preferred social media platform for enjoyment (e.g., Facebook, Instagram, Twitter, Snapchat, Pinterest, Other—please specify). Desire thinking, metacognitions, passive versus active use, and problematic social media use were measured using the following scales.

#### 6.2.1 Active and Passive Social Media Use

A seven‐item scale assessing active and passive use was adopted from Escobar‐Viera et al. (2018), based on Pagani et al. (2011), and validated by Li (2016). Items were rated on a 5‐point Likert‐type scale from 1 (*never*) to 5 (*very frequently*). Four items assessed active use (e.g., “*how often did you post your own content*”), and three assessed passive use (e.g., “*how often did you watch videos or view pictures”*). Construct reliability in the original model was excellent (active CR ranged from 0.983 to 0.990, passive CR = 0.991). Internal reliability in Escobar‐Viera et al. (2018) ranged from good (active *α* = 0.80) to acceptable (passive *α* = 0.72), and in this study from good (active *α* = 0.84) to acceptable (passive *α* = 0.73; Kline 2000).

#### 6.2.2 Bergen Social Media Addiction Scale (BSMAS; Andreassen et al. 2017)

The BSMAS is a six‐item scale assessing PSMU. Items were rated on a 5‐point Likert‐type scale from 1 (*very rarely*) to 5 (*very often*). The BSMAS aligns with the diagnostic and statistical manual of mental disorders (5th ed., text rev.) (American Psychiatric Association 2022) addiction criteria (i.e., salience, mood, modification, tolerance, withdrawal, conflict, and relapse; e.g., “How often during the last year have you become restless or troubled if you have been prohibited from using social media?”). Higher scores indicate greater potential for PSMU. The psychometric validity of the BSMAS scale is well‐established (Bányai et al. 2017). Internal reliability was good (*α* = 0.88; Andreassen et al. 2017) and excellent (*α* = 0.91) in this study.

#### 6.2.3 Desire Thinking Questionnaire (DTQ; Caselli and Spada 2011)

The DTQ is a 10‐item scale assessing IP and VP. IP measures visual elaboration of thoughts about social media (five items: e.g., “*I imagine myself using social media*”), while VP assesses verbal elaboration (five items: e.g., *“I repeat mentally to myself that I need to use social media*”). Items were rated on a 4‐point forced Likert‐type scale ranging from 1 (*almost never*) to 4 (*almost always*), with higher scores indicating increased desire thinking. The original scale's internal reliability was good (total DTQ *α* = 0.83) to acceptable (DTVP and DTIP subscales *α* = 0.78). Moreover, Wilcoxon Signed Ranks tests revealed no significant change in mean factor scores over an 8‐week interval, suggesting stable characteristics of the DTQ (Caselli and Spada 2011). In this study, internal reliability was good (total DTQ *α* = 0.81) to acceptable (DTVP *α* = 0.72) and questionable (DTIP *α* = 0.68).

#### 6.2.4 Metacognitions in Desire Thinking (MDTQ; Caselli and Spada 2013)

The MDTQ is an 18‐item scale measuring metacognitions about desire thinking. Items were rated on a 4‐point forced Likert‐type scale from 1 (*do not agree*) to 4 (*agree very much*). The MDTQ assesses three dimensions: positive metacognitions about desire thinking (PMDT; eight items; e.g., “I need to think about my favourite social media platform in order to feel motivated”), negative metacognitions about desire thinking (NMDT; six items; e.g., “when I begin thinking about using social media I cannot stop”), and the need to control metacognitions about desire thinking (NCDT). Higher scores indicate increased metacognition levels. The original scale's internal reliability was good (total MDTQ *α* = 0.81) to acceptable (PMDT *α* = 0.79, NMDT *α* = 0.75), and demonstrates robust test–retest reliability and stability over an 8‐week interval, with significant correlations for PMDT (*r* = 0.65), NMDT (*r* = 0.65), and NCDT (*r* = 0.68) (Caselli and Spada 2013). This study's internal reliability coefficients were excellent (total MDTQ *α* = 0.95, PMDT *α* = 0.93, NMDT *α* = 0.91). NCDT data were collected but omitted from the final model, following previous research (Caselli and Spada 2015).

### 6.3 Procedure

A university human research ethics committee granted ethical approval. This cross‐sectional study used an anonymous online self‐report survey, with convenience sampling via Facebook and Reddit for recruitment. Before starting, participants were informed about the research purpose, risks, and benefits. After accessing the survey link, participants reviewed the Research Project Information Sheet (RPIS), acknowledging the requirements of being ≥ 18 years of age and able to comprehend English. Consent was implied by progression through the survey, and participants could choose to enter a draw for two $50 Amazon vouchers for survey completion.

### 6.4 Statistical Analysis

Analyses were conducted using SPSS and AMOS (v29). Path analysis was used to assess the relationships among variables and model fit. This was followed by a multigroup analysis aimed at distinguishing potential differences in the patterns of associations between variables across the designated groups. Post hoc analyses examined the direct path specifically between Instagram use and NMDT. The significance of both direct and indirect pathways was also evaluated by generating 10,000 bootstrap samples with confidence intervals, congruent with the bootstrap methods proposed by Shrout and Bolger (2002).

To assess model fit, the Chi‐square (χ2) statistic, Comparative Fit Index (CFI), Standardized Root Mean Square Residual (SRMR), and Root Mean Square Error of Approximation (RMSEA) were utilized. A non‐significant Chi‐square value indicates good fit. CFI values ≥ 0.95 suggest a good fit, while values between 0.90–0.95 indicate an acceptable fit. SRMR values ≤ 0.08 indicate a good fit. RMSEA values ≤ 0.06 represent a good fit, and values between 0.06 and 0.08 are considered acceptable. These indices comprehensively evaluate the model's fit to the data, ensuring reliable and valid results (Kline 2015).

## 7 Results

### 7.1 Data Screening

Of the 466 received responses, complete case analysis removed 129, resulting in a final sample size of *N* = 337. Manifest variables were screened for path analysis assumptions. Most variables were normally distributed except metacognitions about desire thinking, with slight positive skew. Two outliers were identified but did not significantly impact the data (Tabachnick and Fidell 2021). No concerns were identified regarding linearity, multicollinearity, and homoscedasticity. Participants were grouped based on their preferred use of a platform: Facebook (*n* = 131) and Instagram (*n* = 133). For the second analysis, assessing passive use without concurrent active use, participants were separated into two groups based on mean scores, focusing primarily on passive or active use. The predominantly passive group (*N* = 92) scored below the mean on active measures (11.07) and above the mean on passive measures (9.97). The predominantly active group (*N* = 245) scored above the mean on active measures or below the mean on both active and passive measures. This approach was supported by Escobar‐Viera et al. (2018), from which the active and passive scale was adopted.

Minimum sample size recommendations typically depend on the ratio of sample size to the estimated parameters, with suggested ratios varying from 5:1 to 20:1 (Kline 2015). Thus, a minimum sample size for a model with 12 parameters would range from 60 to 240. G*Power software (Faul et al. 2009) was also used as an approximation, in line with Hair et al. (2021)'s recommendations. Preliminary analysis with a medium effect size (0.15) yielded a minimum suggested sample size of 127 for SEM, while a large effect size (0.35) required a sample size of 61. These estimates echo the N:q ratio‐based recommendations, indicating that care must be taken interpreting differences between the active (*N* = 245) and passive (*N* = 92) groups. Importantly, Kline (2015) notes that sample size exerts less influence in well‐fitting models, suggesting that sample size considerations may be less crucial pending model fit outcomes.

**FIGURE 2:**
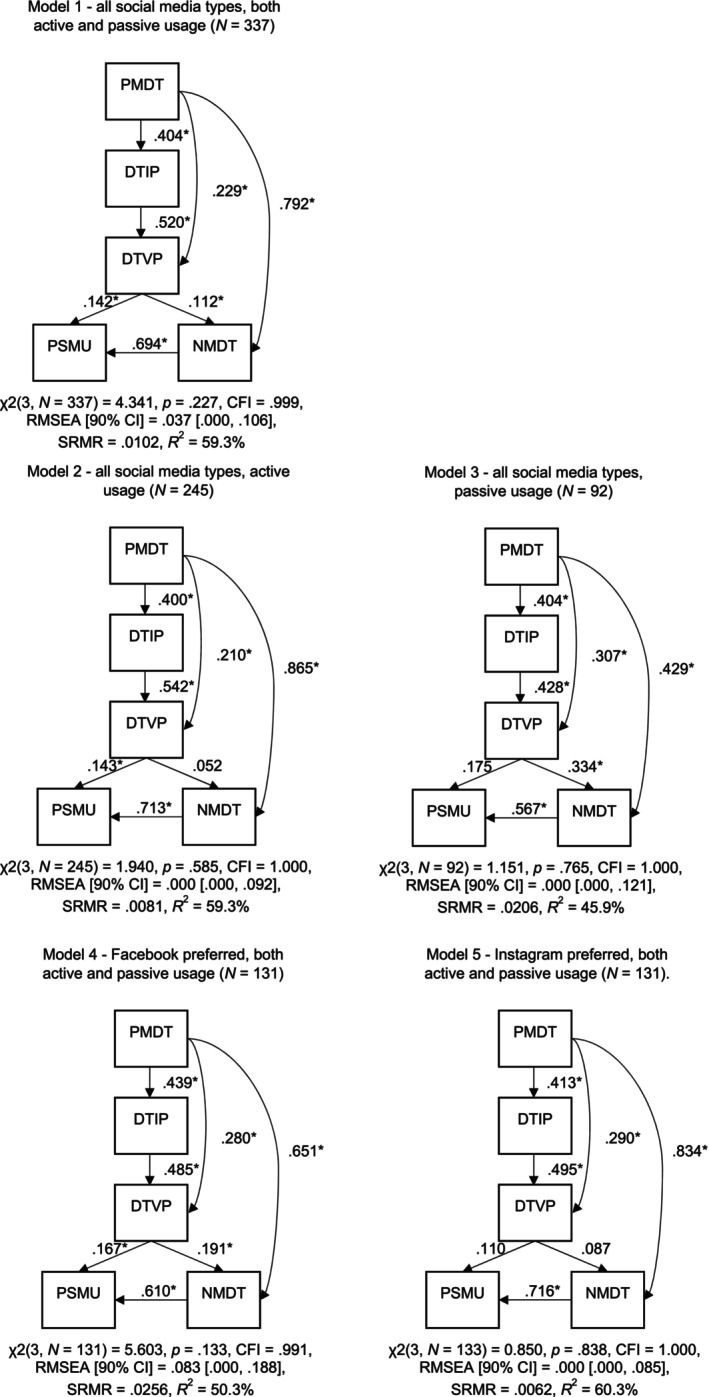
Standardized regression weights, fit indices, and PSMU variance explained for the different models. CFI, comparative fit index; *R* ^2^, variance explained; RMSEA, root mean square error of approximation; SRMR, standardized root mean square residual; χ^2^, Chi‐square statistics. *p < 0.05 (2‐tailed).

For all models, the model fit and PSMU variance explained are summarized in Figure 2. Table 1 shows the direct pathways. Table 2 comprises all studied models' correlations, means, and standard deviations. The fit indices for the models following the exclusion of non‐significant pathways are presented in Table 3. Meanwhile, Table 4 examines indirect effects and confidence intervals (CIs).

**TABLE 1:** Direct pathways for each model (standardized regression weights).

Pathway	Model 1	Model 2	Model 3	Model 4	Model 5
PMDT ➔ DTIP	0.404*	0.400*	0.404*	0.439*	0.413*
PMDT ➔ DTVP	0.229*	0.210*	0.307*	0.280*	0.290*
PMDT ➔ NMDT	0.792*	0.865*	0.429*	0.651*	0.834*
DTIP ➔ DTVP	0.520*	0.542*	0.428*	0.485*	0.495*
DTVP ➔ NMDT	0.112*	0.052	0.334*	0.191*	0.087
DTVP ➔ PSMU	0.142*	0.143*	0.175	0.167*	0.110
NMDT ➔ PSMU	0.694*	0.713*	0.567*	0.610*	0.716*

*Note:* Model 1 = All social media types, both active and passive usage (*N* = 337); Model 2 = All social media types, active usage only (*N* = 245); Model 3 = All social media types, passive usage only (*N* = 92); Model 4 = Facebook preferred, both active and passive usage (*N* = 131); Model 5 = Instagram preferred, both active and passive usage (*N* = 131).

Abbreviations: DTIP, desire thinking imaginal prefiguration; DTVP, desire thinking verbal perseveration; NMDT, negative metacognitions about desire thinking; PMDT, positive metacognitions about desire thinking; PSMU, problematic social media use.

* *p* < 0.05 (2‐tailed).

**TABLE 2:** Descriptive and correlation analysis of hypothesized variables.

Variable	*M(SD)*	1	2	3	4	5
Hypothesis 1: All social media types, both active and passive usage (*N* = 337)						
1. PMDT	13.45 (5.87)	—				
2. NMDT	10.91 (4.65)	0.841**	—			
3. DTIP	12.76 (4.28)	0.404**	0.376**	—		
4. DTVP	12.93 (4.62)	0.440**	0.460**	0.612**	—	
5. PSMU	14.64 (5.97)	0.684**	0.760**	0.358**	0.461**	—
Hypothesis 2: All social media types, active usage (*N* = 245)						
1. PMDT	14.50 (6.28)	—				
2. NMDT	11.44 (4.65)	0.888**	—			
3. DTIP	13.07 (4.26)	0.400**	0.374**	—		
4. DTVP	13.30 (4.61)	0.427**	0.421**	0.626**	—	
5. PSMU	15.26 (6.25)	0.715**	0.773**	0.382**	0.444**	—
Hypothesis 2: All social media types, passive usage (*N* = 92)						
1. PMDT	10.64 (3.22)	—				
2. NMDT	9.51 (3.89)	0.590**	—			
3. DTIP	11.92 (4.24)	0.404**	0.331**	—		
4. DTVP	11.95 (4.53)	0.480**	0.540**	0.552**	—	
5. PSMU	13.00 (4.80)	0.435**	0.661**	0.227**	0.481**	—
Hypothesis 3: Facebook‐preferred, both active and passive usage (*N* = 131)						
1. PMDT	12.91 (5.13)	—				
2. NMDT	10.51 (3.91)	0.745**	—			
3. DTIP	11.99 (4.33)	0.439**	0.397**	—		
4. DTVP	12.86 (4.60)	0.493**	0.512**	0.608**	—	
5. PSMU	14.04 (5.54)	0.632**	0.695**	0.373**	0.479**	—
Hypothesis 3: Instagram‐preferred, both active and passive usage (*N* = 133)						
1. PMDT	13.64 (6.31)	—				
2. NMDT	11.11 (5.15)	0.877**	—			
3. DTIP	13.23 (4.39)	0.413**	0.401**	—		
4. DTVP	13.08 (4.63)	0.495**	0.499**	0.615**	—	
5. PSMU	14.92 (6.27)	0.705**	0.771**	0.347**	0.467**	—

Abbreviations: DTIP, desire thinking imaginal prefiguration; DTVP, desire thinking verbal perseveration; NMDT, negative metacognitions about desire thinking; PMDT, positive metacognitions about desire thinking; PSMU, problematic social media use.

** *p* < 0.01 (2‐tailed).

**TABLE 3:** Fit indices and PSMU variance explained for the different models.

Model	*χ* ^2^	CFI	SRMR	RMSEA [90% CI]	*R* ^2^
Hypothesis 1: All social media types, both active and passive usage (*N* = 337)					
Original model	*χ* ^2^ (3, *N* = 337) = 4.341, *p* = 0.227	0.999	0.0102	0.037 [0.000, 0.106]	59.3%
Hypothesis 2: All social media types, active usage (*N* = 245)					
Original model	*χ* ^2^ (3, *N* = 245) = 1.940, *p* = 0.585	1.000	0.0081	0.000 [0.000, 0.092]	59.3%
Removal of non‐significant pathway	*χ* ^2^ (4, *N* = 245) = 4.441, *p* = 0.350	0.999	0.0177	0.021 [0.000, 0.102]	61.1%
Hypothesis 2: All social media types, passive usage (*N* = 92)					
Original model	*χ* ^2^ (3, *N* = 92) = 1.151, *p* = 0.765	1.000	0.0206	0.000 [0.000, 0.121]	45.9%
Removal of non‐significant pathway	*χ* ^2^ (4, *N* = 92) = 4.607, *p* = 0.330	0.996	0.0348	0.042 [0.000, 0.171]	43.7%
Hypothesis 3: Facebook‐preferred, both active and passive usage (*N* = 131)					
Original model	*χ* ^2^ (3, *N* = 131) = 5.603, *p* = 0.133	0.991	0.0256	0.083 [0.000, 0.188]	50.3%
Hypothesis 3: Instagram‐preferred, both active and passive usage (*N* = 133)					
Original model	*χ* ^2^ (3, *N* = 133) = 0.850, *p* = 0.838	1.000	0.0062	0.000 [0.000, 0.085]	60.3%
Removal of non‐significant pathways	*χ* ^2^ (5, *N* = 133) = 6.968, *p* = 0.223	0.995	0.0439	0.055 [0.000, 0.143]	59.4%

Abbreviations: *χ* ^2^, Chi‐square statistics; CFI, comparative fit index; *R* ^2^, variance explained; RMSEA, root mean square error of approximation; SRMR, standardized root mean square residual.

**TABLE 4:** Examination of indirect effects and 95% confidence intervals (CIs).

Indirect pathways	Standardized indirect effects	Unstandardized bootstrap estimate		95% CI		Significance
	*β*	*B*	SE	Lower	Upper	
Hypothesis 1: All social media types, both active and passive usage (*N* = 337)						
PMDT>DTIP>DTVP>NMDT>PSMU	0.016	0.017	0.005	0.007	0.029	0.000
PMDT>DTVP>NMDT>PSMU	0.017	0.018	0.007	0.008	0.033	0.000
PMDT>NMDT>PSMU	0.545	0.559	0.039	0.480	0.634	0.000
PMDT>DTVP>PSMU	0.032	0.033	0.011	0.016	0.062	0.000
DTIP>DTVP>NMDT>PSMU	0.039	0.056	0.016	0.026	0.091	0.000
DTIP>DTVP>PSMU	0.073	0.103	0.028	0.052	0.162	0.000
DTVP>NMDT>PSMU	0.076	0.100	0.029	0.045	0.159	0.000
Hypothesis 2: All social media types, active usage (*N* = 245)						
PMDT>DTIP>DTVP>NMDT>PSMU	0.008	0.008	0.005	0.000	0.020	0.061
PMDT>DTVP>NMDT>PSMU	0.007	0.008	0.005	0.000	0.022	0.059
PMDT>NMDT>PSMU	0.618	0.613	0.042	0.528	0.695	0.000
PMDT>DTVP>PSMU	0.029	0.030	0.012	0.012	0.060	0.000
DTIP>DTVP>NMDT>PSMU	0.019	0.029	0.017	−0.003	0.065	0.073
DTIP>DTVP>PSMU	0.076	0.114	0.034	0.052	0.186	0.000
DTVP>NMDT>PSMU	0.036	0.050	0.030	−0.0060	0.112	0.079
Hypothesis 2: All social media types, passive usage (*N* = 92)						
PMDT>DTIP>DTVP>NMDT>PSMU	0.032	0.049	0.023	0.019	0.116	0.000
PMDT>DTVP>NMDT>PSMU	0.058	0.087	0.033	0.040	0.177	0.000
PMDT>NMDT>PSMU	0.245	0.363	0.133	0.130	0.654	0.001
PMDT>DTVP>PSMU	0.053	0.080	0.046	0.006	0.190	0.032
DTIP>DTVP>NMDT>PSMU	0.081	0.092	0.035	0.040	0.183	0.000
DTIP>DTVP>PSMU	0.073	0.085	0.046	0.006	0.185	0.035
DTVP>NMDT>PSMU	0.188	0.201	0.064	0.096	0.350	0.000
Hypothesis 3: Facebook‐preferred, both active and passive usage (*N* = 131)						
PMDT>DTIP>DTVP>NMDT>PSMU	0.024	0.027	0.013	0.008	0.060	0.002
PMDT>DTVP>NMDT>PSMU	0.030	0.035	0.019	0.010	0.087	0.002
PMDT>NMDT>PSMU	0.397	0.429	0.071	0.295	0.574	0.000
PMDT>DTVP>PSMU	0.048	0.050	0.026	0.013	0.119	0.006
DTIP>DTVP>NMDT>PSMU	0.056	0.072	0.031	0.022	0.147	0.003
DTIP>DTVP>PSMU	0.082	0.103	0.045	0.028	0.205	0.007
DTVP>NMDT>PSMU	0.116	0.140	0.058	0.041	0.270	0.003
Hypothesis 3: Instagram‐preferred, both active and passive usage (*N* = 133)						
PMDT>DTIP>DTVP>NMDT>PSMU	0.013	0.013	0.008	0.001	0.033	0.038
PMDT>DTVP>NMDT>PSMU	0.019	0.018	0.011	0.001	0.046	0.036
PMDT>NMDT>PSMU	0.598	0.593	0.063	0.456	0.706	0.000
PMDT>DTVP>PSMU	0.032	0.032	0.022	−0.001	0.088	0.058
DTIP>DTVP>NMDT>PSMU	0.032	0.044	0.024	0.001	0.099	0.043
DTIP>DTVP>PSMU	0.054	0.078	0.046	−0.006	0.175	0.069
DTVP>NMDT>PSMU	0.065	0.084	0.045	0.000	0.181	0.048

Abbreviations: DTIP, desire thinking imaginal prefiguration; DTVP, desire thinking verbal perseveration; NMDT, negative metacognitions about desire thinking; PMDT, positive metacognitions about desire thinking; PSMU, problematic social media use.

### 7.2 Hypothesis 1

When examining the MCMDT in the collective sample of social media users (Hypothesis 1), the model demonstrated a good fit across all indices, explaining 59.3% of the variance in PSMU. No model modification was required, as all pathways were statistically significant at the 0.05 level. All indirect pathways proved significant, with the 95% confidence intervals not encompassing zero.

### 7.3 Hypothesis 2

The MCMDT for both active and passive social media users displayed a good fit (Hypothesis 2a), explaining 59.3% and 45.9% of the variance in PSMU, respectively. In the model for active users, a non‐significant pathway between DTVP and NMDT (*β* = 0.052, *p* = 0.113) was identified. After removing it, the model fit remained good and explained 61.1% of the variance in PSMU. Similarly, in the passive users model, a non‐significant pathway between DTVP and PSMU (*β* = 0.175, *p* = 0.060) was found. After removing it, the model fit remained good and accounted for 43.7% of the variance in PSMU. The multigroup analysis found no significant differences (Δ*χ* ^2^ = 3.137, Δdf = 6, *p* = 0.791), and all constrained models, which presumed equality of individual variables (PMDT, DTIP, DTVP, NMDT) across groups, offered a similar fit to the unconstrained model.

Aside from two exceptions, the direct pathways were statistically significant for both active and passive users (Hypothesis 2b). The pathway from DTVP to NMDT was non‐significant for active users. In contrast, the pathway from DTVP to PSMU was non‐significant for passive users. Considering indirect pathways (Hypothesis 2b), results varied. For passive users, all indirect pathways were significant, as evidenced by the 95% confidence intervals not encompassing zero. For active users, however, four pathways did not reach significance.

### 7.4 Hypothesis 3

The Facebook‐preferred model (Hypothesis 3a) displayed acceptable fit and explained 50.3% of the variance in PSMU, with all pathways significant at the 0.05 level. In contrast, the Instagram‐preferred model showed the best fit among all models, accounting for 60.3% of the variance in PSMU. However, two non‐significant pathways were identified: DTVP to NMDT (*β* = 0.087, *p* = 0.072) and DTVP to PSMU (*β* = 0.110, *p* = 0.086). After removing these pathways, the model remained a good fit and explained 59.4% of the variance in PSMU. The multigroup analysis found no significant differences (Δχ^2^ = 6.555, Δdf = 6, *p* = 0.364), and the constrained models aligned similarly with the unconstrained model.

Facebook users exhibited a more pronounced VP activation (Hypothesis 3b). This was evident in the significant outcomes for all direct and indirect VP‐related pathways. For Instagram users, the VP‐related pathways from DTVP to NMDT and from DTVP to PSMU were not significant. Instagram users did not exhibit enhanced IP activation. Notably, the Facebook‐preferred model demonstrated IP activation, as evidenced by significant results across both direct and indirect pathways linked to IP. In contrast, the activation of IP on Instagram exhibited a more complex pattern, with one IP‐related indirect pathway failing to attain statistical significance, and two pathways encompassing zero within their 95% confidence intervals.

To gain more insight into the unexpected patterns of IP activation among Instagram users, a post hoc analysis was conducted. This additional analysis examined a potential direct path between IP and NMDT in the Instagram user group. However, the direct path was found to be non‐significant at the 0.005 level, suggesting no relationship between DTIP and NMDT for Instagram users in this sample.

## 8 Discussion

This study highlights the transdiagnostic value of the MCMDT in understanding PSMU, confirming the primary elements of the projected model across all models and usage types. Notably, consistent positive relationships were observed between PMDT and both VP and IP across all models, indicating that positive metacognitions prompt mental visualization and verbal rehearsal of social media use. Furthermore, the significant positive association between IP and VP in all models emphasizes increased desire thoughts during the elaboration stage, reinforcing the relationship where IP aids VP in maintaining and amplifying desire thoughts. However, distinctions emerged when examining the pathological amplification of desire thinking stage of the model.

### 8.1 Hypothesis 1

Hypothesis 1 results support all model components, including pathological amplification of desire thinking. The relationships between VP, negative metacognitions about desire thinking, and PSMU were significant and positive, suggesting that desire thinking serves as a cognitive elaboration strategy, amplifying negative metacognitions and affecting PSMU directly and indirectly.

### 8.2 Hypothesis 2

The significant relationship between VP and negative metacognitions about desire thinking for passive users, but not for active users, was unexpected. Research suggests that if a non‐significant relationship were to be observed, it should have been for passive users, as their behavior typically involves less active cognitive engagement (Verduyn et al. 2015). Passive social media use, characterized by activities like mindlessly scrolling through feeds or watching videos without much conscious thought or engagement, often exhibits patterns of automatic and repetitive behavior (Verduyn et al. 2015). Gardner et al. (2012) propose that such behaviors can bypass conscious thought, leading to reduced metacognitive awareness. One potential explanation for the activation of negative metacognitions in passive users is the lower cognitive load associated with their style of social media use. Passive engagement may leave cognitive resources available for self‐referential processing and repetitive thought patterns (Verduyn et al. 2015), which contribute to the development of negative metacognitive beliefs through Cognitive Attentional Syndrome (Wells, 2009). Another possibility is that passive users may encounter heightened perceived social comparison or feelings of inadequacy, driving negative metacognitions (Verduyn et al. 2015). Indeed, research indicates that passive social media users can be more prone to anxiety, worry, and rumination, possibly due to increased social comparison, feelings of isolation, and harm avoidance (Frison and Eggermont 2020; Verduyn et al. 2015). The non‐significant relationship in active users was unanticipated. Constant cognitive engagement and anticipation of rewards from social media may also make users less susceptible to negative metacognitions, as they focus more on the potential gains and satisfaction derived from their online experiences, consistent with a reward‐focused cognitive loop (Caselli and Spada 2015).

The relationship between VP and PSMU was significant for active users but non‐significant for passive users. Active users, who are more immersed in the cognitive processing of their online experiences, may experience a direct drive from VP to PSMU as they constantly evaluate and plan their online interactions. In contrast, the absence of association between VP and PSMU for passive users could again be attributed to their lower cognitive load and diminished engagement in the reward‐driven processes associated with active social media use. Passive users might not receive the same level of reinforcement from their online experiences. As a result, the desire to use social media may not be as strongly influenced by VP as it is for active users. Similarly, habit‐formation models propose that low‐frequency, low‐effort behaviors can become automatic over time, reducing the need for active engagement (Garnder et al., 2012).

### 8.3 Hypothesis 3

The relationship between VP and negative metacognitions about desire thinking and the relationship between VP and PSMU were both significant for Facebook‐preferred users but non‐significant for Instagram‐preferred users. The results for Facebook were consistent with the hypothesized model. Facebook's nature may create more opportunities for VP as users engage in extensive internal dialogue about their experiences, analyzing past interactions, and planning future ones. As hypothesized, this heightened VP leads to increased negative metacognition and ultimately contributes to PSMU.

However, Instagram, primarily a visual platform, displayed different patterns of desire thinking activation. While the study hypothesized that Instagram would exhibit a stronger influence of IP than VP due to its image‐centric nature, the findings did not support this expectation. Instagram users demonstrated a more complex pattern of IP activation, with certain pathways failing to attain statistical significance. This suggests that while VP may have a reduced impact on Instagram users regarding negative metacognitions and PSMU, IP does not necessarily fill this role as anticipated. It is plausible that Instagram users might engage in self‐regulatory strategies before reaching the pathological amplification of desire thinking, thereby preventing an overt escalation of PSMU. The consistently higher levels of IP than VP across all models support this idea, suggesting that users may rely more on imaginal cognitive strategies to regulate their internal states when using social media platforms. Spada et al. (2015) found that this distinction allowed individuals, particularly those who binge ate, to regulate their internal states better.

Caselli and Spada et al. (2015) observed a similar phenomenon in their original community studies, suggesting that escalating distress might not necessarily lead to negative metacognitions about the uncontrollability of desire thoughts. This aligns with Mansueto et al.'s (2019) systematic review, which found a stronger association between VP and physical addictions, but not necessarily IP. Negative metacognitions about desire thinking may play a lesser role in escalating PSMU in a community sample, as non‐clinical individuals may not experience the same level of distress or perceived uncontrollability.

Across all models, a direct relationship was observed between positive and negative metacognitions about desire thinking. This observation departs from the original theoretical expectations of the MCMDT, which proposed that such a relationship should be indirect, mediated by cognitive elaboration strategies. Nevertheless, these findings align with Caselli and Spada's (2015) original studies, suggesting a more complex interplay between positive and negative metacognitions than initially anticipated.

While this study offers valuable insights, it has several limitations. First, the adult sample may limit the generalizability of the findings to other age groups, particularly teenagers, who are most at‐risk for PSMU (Kuss and Griffiths 2017). Future research should investigate more diverse samples. Second, the cross‐sectional design prevents conclusions about causality; longitudinal and experimental designs should be utilized in future research. Third, the operationalization of active and passive use may not accurately represent these constructs, needing improvement in future studies. Fourth, the categorization of Facebook and Instagram groups was based on preference and did not account for multi‐platform use. Lastly, while the sample sizes for sub‐group analyses adhered to basic guidelines, they were not extensive. However, the robust fit of the models compensates, to some extent, for this shortcoming, reinforcing the statistical validity of these findings. Building on this study's promising and novel findings, future research could expand the sample size to replicate the results and allow more detailed comparisons across user types and platforms. Larger sampling may also allow for more accurate subgrouping by standardizing the mean.

In conclusion, this study offers valuable insights with significant implications for research and clinical practice. The findings reinforce earlier research, validating the MCMDT's robustness and applicability across behavioral addictions and establishing its relevance to social media addictions. Moreover, this study emphasizes the importance of considering platform‐specific nuances and the interplay between passive and active social media use. These insights highlight the potential benefits of addressing metacognitive factors in interventions designed to reduce problematic social media use and enhance well‐being. While the understanding of how desire thinking components (VP and IP) interact with and influence the subsequent stages of the metacognitive model is less clear‐cut than initially postulated, especially concerning specific social media platforms, this study lays groundwork for future explorations into the cognitive and metacognitive dynamics of social media use. By investigating these relationships in various populations and contexts, researchers can contribute to developing more effective, tailored interventions for those struggling with problematic social media use, promoting healthier engagement with social media platforms and improved overall well‐being.

## Author Contributions

G.S.W., A.A., and L.K.‐D. contributed to the study's conception and design. A.K. performed material preparation and data collection. G.S.‐W. and A.A. performed data analysis. G.S.‐W. wrote the first draft of the manuscript. A.A. and L.K.‐D. commented on previous versions of the manuscript. All authors read and approved the final manuscript.

## Disclosure

The authors have nothing to report.

## Ethics Statement

The questionnaire and methodology for this study were approved by the Human Research Ethics committee of the University of the Sunshine Coast Ethics approval number: (S201483).

## Consent

Informed consent was obtained from all individual participants included in the study.

## Conflicts of Interest

The authors declare no conflicts of interest.

## Data Availability

The data that support the findings of this study are available from the corresponding author upon reasonable request.
